# Psychometric Properties of the Quick Inventory of Depressive Symptomatology-Self-Report (QIDS-SR) in Depressed Adolescents

**DOI:** 10.3389/fpsyt.2020.598609

**Published:** 2020-12-09

**Authors:** Wu-Yang Zhang, Yan-Jie Zhao, Yao Zhang, Fan He, Hong-Qing Pan, Teris Cheung, Gabor S. Ungvari, Shu-Ying Li, Yu-Tao Xiang

**Affiliations:** ^1^Department of Psychiatry, The First Affiliated Hospital of Zhengzhou University, Zhengzhou, China; ^2^Unit of Psychiatry, Institute of Translational Medicine, Faculty of Health Sciences, University of Macau, Macao, China; ^3^Center for Cognition and Brain Sciences, University of Macau, Macao, China; ^4^Institute of Advanced Studies in Humanities and Social Sciences, University of Macau, Macao, China; ^5^The National Clinical Research Center for Mental Disorders, Beijing Key Laboratory of Mental Disorders Beijing Anding Hospital, The Advanced Innovation Center for Human Brain Protection, School of Mental Health, Capital Medical University, Beijing, China; ^6^School of Nursing, Hong Kong Polytechnic University, Hong Kong, China; ^7^Division of Psychiatry, School of Medicine, University of Western Australia/Graylands Hospital, Perth, WA, Australia; ^8^Department of Psychiatry, University of Notre Dame Australia, Fremantle, WA, Australia

**Keywords:** depression, reliability, validity, C-QIDS-SR, adolescents

## Abstract

**Background:** Accurate and reliable self-report measurement tools examining depressive symptoms are scant in child psychiatry. This study assessed the psychometric properties of the Chinese Version of the Quick Inventory of Depressive Symptomatology-Self-Report (C-QIDS-SR) in depressed adolescents in China.

**Methods:** Adolescents with major depressive episodes or bipolar depressive episodes were assessed using the C-QIDS-SR. The structure validity of the C-QIDS-SR was estimated using principal component analysis with varimax rotation.

**Results:** A total of 246 depressed adolescents were included in the study. Cronbach's alpha was 0.77. The correlation coefficient between the baseline and endpoint assessments was 0.49 (*p* < 0.001), whereas, the correlation coefficient between the C-QIDS-SR and the Hamilton Rating Scale for Depression−17 items (HAMD-17) was 0.63 (*p* < 0.001). Principal component analysis with varimax rotation demonstrated the unidimensional structure of the C-QIDS-SR.

**Conclusion:** The C-QIDS-SR is a reliable and valid instrument with acceptable psychometric properties to measure depressive symptoms in adolescents.

## Introduction

Depression is common in adolescents (1). The National Comorbidity Survey–Adolescent Supplement (NCS-A) found that the lifetime and 12-months prevalence estimates of major depression in adolescents in the US were 11.0 and 7.5%, respectively (2). Depression in adolescents is associated with a range of adverse outcomes, such as cognitive decline, impaired resilience and suicidal ideation (3, 4). In the past few decades, standardized measurement tools have been developed and adopted to measure depressive symptoms in clinical practice and research for both adult and adolescent patients, such as the Hamilton Rating Scale for Depression (HAMD) (5) and the Montgomery-Asberg Depression Rating Scale (MADRS) (6). However, these scales are time-consuming and have to be administered by clinicians or trained researchers (7). Therefore, user-friendly, accurate, and short measurement tools on self-assessment of depressive symptoms are warranted (8, 9). Several self-rating measures have been developed, such as the Patient Health Questionnaire-9 (PHQ-9) (10, 11), the Beck Depression Inventory (BDI) (12, 13), and the Self-Rating Depression Scale (SDS) (14, 15).

The 16-item Quick Inventory of Depressive Symptomatology-Self-Report (QIDS-SR) is a widely used instrument with satisfactory psychometric properties (16). The 16 items of the QIDS-SR were derived from the nine symptom criteria for major depressive disorder (MDD) in the Diagnostic and Statistical Manual of Mental Disorders, fourth edition (DSM-IV) (17). This scale assesses the severity of depressive symptoms in patients with MDD or bipolar disorder (BP) (18). The Chinese version of QIDS-SR was introduced in China in 2012 and it has proven to be a reliable and valid tool in adult patients with MDD (19) and other major psychiatric disorders and major medical conditions, such as schizophrenia (20), and hepatitis B virus (HBV)-related liver disease (21).

In 2015 there were around 271 million children and adolescents (<18 years old) in China; 85 million of them were adolescents (12–17 years) (22). It is estimated that 19.85% of Chinese children and adolescents suffer from depressive symptoms (23), which means that around 16.9 million adolescents require timely assessment and treatment. Yet, as far as we could ascertain, to date very few studies have examined the usefulness of standardized instruments in depressed adolescents, which gave the impetus to investigate the psychometric properties of the QIDS-SR in adolescents with MDD or BP.

## Methods

### Settings and Subjects

This study was conducted in Department of Child Psychiatry, the First Affiliated Hospital of Zhengzhou University, China. Patients who met the following inclusion criteria were consecutively invited to participate in the study: (1) adolescents aged between 12 and 17 years; (2) were diagnosed with major depressive episodes (MDE) or bipolar depressive episodes (depressed adolescents hereafter) according to the International Statistical Classification of Diseases and Related Health Problems, 10th revision (ICD-10) by a review of medical records; (3) scored a total of ≥7 in the Hamilton Rating Scale for Depression−17 items (HAMD-17); and (4) were able to understand the purpose and contents of the assessment. Patients with cognitive impairment or intellectual disability were excluded. The study protocol was approved by the Institutional Review Board (IRB) of the First Affiliated Hospital of Zhengzhou University. All participants provided oral informed consent whilst their legal guardians provided written informed consent prior to participation in the study.

### Assessment Instrument

Basic demographics and clinical characteristics were collected using a form designed for this study. The Chinese version of the Quick Inventory of Depressive Symptomatology-Self-rating (C-QIDS-SR) was used to measure the severity of depressive symptoms during the past week. The C-QIDS-SR consists of 16 items, in nine domains: (1) sleep; (2) sad mood; (3) appetite/weight change; (4) concentration/decision making; (5) self-outlook; (6) thoughts of death or suicide; (7) involvement; (8) energy level; and (9) agitation/retardation (16, 19). The C-QIDS-SR total score ranges from 0 to 27, with higher scores indicating more severe depressive symptomatology.

The HAMD-17 was used to assess the severity of depressive symptoms in the past week. The Chinese version of the HAMD-17 has been validated in Chinese populations yielding good psychometric properties (5, 24). The HAMD-17 was applied to screen depressed adolescents and assess criterion-related validity in this study. Participants were interviewed by a child psychiatrist who received formal training in the administration of HAMD-17.

### Statistical Analysis

Data were analyzed with the Statistical Analysis System, University Edition (SAS Institute Inc., Cary, North Carolina, U.S.). Internal consistent reliability was examined with the Cronbach's alpha coefficient: an alpha of 0.6 or higher was considered acceptable (20, 25). The associations between C-QIDS-SR total score and domain scores, and test-retest reliability were examined by the aid of Pearson's correlation coefficient. Criterion validity was assessed with Pearson's correlation coefficient between C-QIDS-SR and HAMD-17 total scores. The structure validity of the C-QIDS-SR in depressed adolescents was estimated by exploratory factor analysis. A principal component analysis (PCA) with varimax rotation was performed to extract the factors and obtain the most meaningful original factor structure of the C-QIDS-SR. If one factor explained 20% or more of total variance, the scale was considered to be unidimensional (20, 26, 27). Level of significance level was set at <0.05 (two-tailed).

## Results

A total of 246 depressed adolescents were recruited in this study; 170 were diagnosed with MDE and the rest with bipolar depressive episode. The basic demographic and clinical characteristics of the sample are shown in Table 1.

**Table 1:** Basic demographics and clinical characteristics of the study sample.

	**Whole sample (*****N*** **= 246)**	
	***N***	**%**
Male	90	36.59
**Diagnosis**		
Bipolar disorder	76	30.89
Major depression	170	69.11
Family history of psychiatric disorders	28	11.38
	**Mean**	***SD***
Age (years)	15.16	1.42
Age of onset (years)	13.75	1.95
Education level (years)	9.05	1.55
Number of episodes	1.49	0.90
HAMD-17 total score	35.17	12.60

*HAMD-17, Hamilton Rating Scale for Depression −17 items*.

A Cronbach's alpha of 0.77 indicated satisfactory internal consistency and homogeneity between C-QIDS-SR items. The item-total correlation coefficients in the five domains of sad mood, concentration/decision making, self-outlook, thoughts of death or suicide, energy level of the C-QIDS-SR were above 0.60, demonstrating acceptable item-total correlation. The item-total correlation coefficients were not affected if any of these domains were omitted (Table 2).

**Table 2:** QIDS rating at baseline in depressed Chinese adolescents (*N* = 246).

	**Mean**	***SD***	**Item-total correlation**	**Alpha if item deleted**
1. Sleep	2.18	0.78	0.44^**^	0.77
2. Sad mood	1.72	0.75	0.65^**^	0.74
3. Appetite/weight change	1.76	0.99	0.51^**^	0.76
4. Concentration/Decision making	1.64	0.9	0.72^**^	0.72
5. Self-outlook	1.87	1.02	0.65^**^	0.74
6. Thoughts of death or suicide	1.64	1.03	0.65^**^	0.74
7. Involvement	1.62	0.94	0.59^**^	0.75
8. Energy level	1.37	1.15	0.60^**^	0.75
9. Agitation/retardation	1.72	0.96	0.55^**^	0.75
Total score	15.49	5.11	—	—

** *p < 0.01*.

To examine the test-retest reliability, 91 participants, received the C-QIDS-SR assessment twice within 2 weeks. The Pearson's correlation coefficient between baseline and endpoint assessments was 0.49 (*p* < 0.001), showing moderate test-retest reliability. The correlation coefficient between the C-QIDS-SR and the HAMD-17 was 0.63 (*p* < 0.001), indicating an acceptable criterion validity.

**Figure 1:**
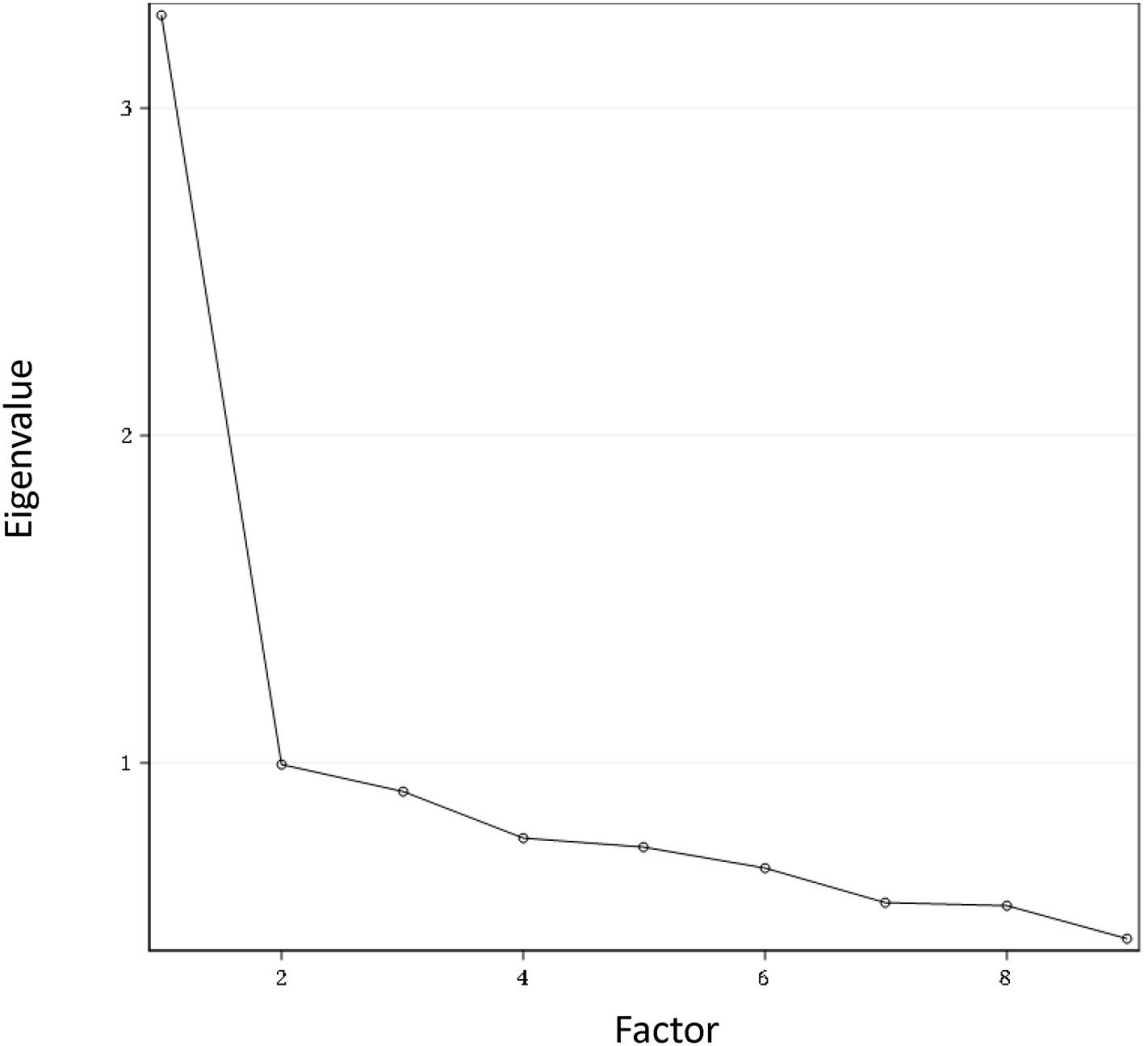
Scree plot for the QIDS-SR at baseline.

Principal component analysis with varimax rotation was performed to examine the structure validity of the C-QIDS-SR. The first factor explained 36.47% of the total variance and being above 20% it signifies that the scale was unidimensional. Figure 1 shows the scree plot with the magnitude of eigenvalue as the function of factor extraction order.

## Discussion

To the best of our knowledge, this was the first study that examined the psychometric properties of C-QIDS-SR in depressed adolescents. C-QIDS-SR was found to be a reliable and valid measurement tool in assessing the severity of depressive symptoms in depressed adolescents. All participants were able to complete this self-reported scale, indicating that the C-QIDS-SR may be an easy-to-use tool in child and adolescent psychiatry.

Internal consistency of the C-QIDS-SR in depressed adolescents (0.77) is similar to the figures found in adult patients with MDD (0.76) (19); either inpatients (0.83) (28), or patients with HBV-related liver diseases (0.796) (21), or depressed schizophrenia patients (0.73) (20). Item-total correlation showed that the “sleep” and “appetite/weight change” domains had the lowest correlation coefficients to the total score (0.44 and 0.51, respectively), which is similar to the findings in adult patients in China (20, 21, 28) and other countries (29). The “concentration/decision making,” “sad mood,” “self-outlook,” “thoughts of death or suicide,” and “energy level” domains had high item-total correlation coefficients (i.e., >0.6), replicating previous findings in adult patients with mood disorders (18).

The test-retest reliability of 0.49 shows acceptable correlation coefficient. The relatively low test-retest reliability might be associated with the fluctuations of depressive symptoms during the 2 weeks of data collection period. Criterion validity for C-QIDS-SR in depressed adolescents was established by comparing C-QIDS-SR with the HAMD-17; the correlation coefficient of 0.63 was higher when compared to studies conducted in adult patients using the HAMD-17 as the reference criterion (both *r* = 0.54) (19, 28). The exploratory factor analysis identified only one principal factor, demonstrating that C-QIDS-SR is a unidimensional scale. All nine domains were closely related to the independent factor, i.e., the overall severity of depression, which is similar to previous findings in China and in other countries (16, 18–21, 28).

Several methodological shortcomings of the study should be addressed. First, participants were only recruited from one major hospital, which limits the generalizability and overall representativeness of the findings. Second, the sample size of adolescents with bipolar depression was relatively small reflecting the actual clinical situation. Third, there were no healthy controls, therefore the discrimination criterion and cutoff value could not be determined. Fourth, the symptomatology of depression in adolescents is somewhat different from that in adults. For example, irritability and anger are more common in depressed adolescents than in adults. The QIDS-SR was originally developed for use in depressed adult populations, thus symptoms more characterized of depressed adolescents were not included in the QIDS-SR, which probably affected its sensitivity in adolescents. Finally, only the principal psychiatric diagnosis were recorded in the electronic medical record system, therefore the impact of comorbidities on the psychometric properties of C-QIDS-SR could not be explored.

In conclusion, C-QIDS-SR has proven to be a reliable, valid, and quick self-report instrument that accurately measured depressive symptoms in adolescents. The psychometric properties of the C-QIDS-SR were acceptable; thus this instrument could be used in both clinical and research settings.

## Data Availability Statement

The original contributions presented in the study are included in the article/supplementary material, further inquiries can be directed to the corresponding author/s.

## Ethics Statement

The studies involving human participants were reviewed and approved by Institutional Review Board (IRB) of the First Affiliated Hospital of Zhengzhou University. Written informed consent to participate in this study was provided by the participants' legal guardian/next of kin.

## Author Contributions

FH, S-YL, and Y-TX: study design. W-YZ, Y-JZ, YZ, and H-QP: data collection, analysis, and interpretation. Y-JZ, TC, and Y-TX: drafting of the manuscript. GU: critical revision of the manuscript. All co-authors: approval of the final version for publication.

## Conflict of Interest

The authors declare that the research was conducted in the absence of any commercial or financial relationships that could be construed as a potential conflict of interest.
